# DnaK Protein Alleviates Toxicity Induced by Citrate-Coated Gold Nanoparticles in *Escherichia coli*

**DOI:** 10.1371/journal.pone.0121243

**Published:** 2015-04-02

**Authors:** Stanley Makumire, Neerish Revaprasadu, Addmore Shonhai

**Affiliations:** 1 Department of Biochemistry, School of Mathematics & Natural Sciences, University of Venda, Thohoyandou, South Africa; 2 Department of Chemistry, University of Zululand, KwaDlangezwa, South Africa; 3 Department of Biochemistry & Microbiology, University of Zululand, KwaDlangezwa, South Africa; New England BioLabs, UNITED STATES

## Abstract

A number of previously reported studies suggest that synthetic gold nanoparticles (AuNPs) are capable of stabilising proteins against heat stress in vitro. However, it remains to be understood if AuNPs confer stability to proteins against cellular stress in vivo. Heat shock proteins (Hsps) are conserved molecules whose main role is to facilitate folding of other proteins (chaperone function). Hsp70 (called DnaK in prokaryotes) is one of the most prominent molecular chaperones. Since gold nanoparticles exhibit chaperone-like function in vitro, we investigated the effect of citrate-coated gold nanoparticles on the growth of *E*. *coli* BB1553 cells that possess a deleted *dnaK* gene. We further investigated the effects of the AuNPs on the solubility of the *E*. *coli* BB1553 proteome. *E*. *coli* BB1553 cells exposed to AuNPs exhibited cellular defects such as filamentation and plasma membranes pulled off the cell wall. The toxic effects of the AuNPs were alleviated by transforming the *E*. *coli* BB1553 cells with a construct expressing DnaK. We also noted that cells in which DnaK was restored exhibited distinct zones to which the nanoparticles were restricted. Our study suggests a role for DnaK in alleviating nanoparticle induced stress in *E*. *coli*.

## Introduction

Gold nanoparticles (AuNPs) are gaining immense attention as biomedical agents because of their compatibility with biological materials [1]. It is thought that upon their introduction to biological environments, AuNPs readily associate with proteins [2]. Proteins attach onto nanoparticle surfaces via several mechanisms such as electrostatic interactions, van der Waals’ forces, hydrophobic, hydrophilic, structural and steric interactions [3]. The modification of nanoparticles through their association with particular proteins influences the functional features of the nanoparticle and/or the attached protein molecule [4]. This results in beneficial effects or undesirable outcomes [5]. Several nanoparticles manifest toxicity based on the nature of their interaction with biological components [6][7][8]. Although gold nanoparticles are fairly inert, there is mounting evidence suggesting that they cause DNA damage [9] and are toxic to bacteria [10][11]. Several studies have reported that gold nanoparticles are readily internalised by bacteria, including *E*. *coli* [10][11]. It has been observed that gold nanoparticles exhibit higher antimicrobial activity in gram negative bacteria as these cells are deemed to internalise the nanoparticles readily [12]. Several mechanisms by which gold nanoparticles cause toxicity in bacteria have been proposed and include collapsing the membrane potential and inhibition of ATPases [13]. It is conceivable that the collapse of the membrane potential promotes leakage of some materials from the cells as well as promoting uptake of the nanoparticles within the vicinity of the cells.

Heat shock proteins (Hsps) are conserved molecules whose main function is to facilitate protein folding [14]. Hsp70 (called DnaK in prokaryotes) is one of the most prominent heat shock proteins [15]. It recognises and binds to hydrophobic patches of misfolded proteins, stabilising them to prevent their aggregation and it is also capable of reversing protein aggregation [16][17]. Because of their role in protein folding, Hsps constitute a major part of the molecular chaperone machinery (facilitators of protein folding) in living systems [18]. Previous studies that we and others conducted suggest that gold nanoparticles (AuNPs) exhibit functional features that resemble those of molecular chaperones [19][20]. We previously observed that gold nanoparticles suppressed the aggregation of heat stressed proteins in vitro [19]. This function seems to be unique for AuNPs as other nanoparticles promote protein aggregation and/or degradation [6][7][21]. It is conceivable that the capability of gold nanoparticles to act as inhibitors of protein aggregation makes them attractive candidates for possible use in the treatment of protein aggregation-induced neurodegenerative diseases as well as protein stabilising agents [19][22].

*E*. *coli* BB1553 (MC4100 Δ*dnaK52*::CmR sidB1) cells lack the *dnaK* gene [23]. The cells grow at an ambient temperature of 30°C, and because of their compromised folding function their growth is inhibited at higher temperatures. *E*. *coli* BB1553 cells have been widely used as a model for the study of protein folding in vivo [24][25]. The same cells have also been used in a study that investigated the effect of antimicrobial agents that target DnaK function [26]. A member of the heat shock protein 70 (Hsp70) family, DnaK is known to enhance the resilience of *E*. *coli* to cell stress [23][27]. Subjecting cells to stress such as heat promotes protein aggregation and one mechanism in which DnaK facilitates cytoprotection against stress is through its capability to prevent and reverse protein misfolding [27]. Consequently, DnaK is over-expressed during cellular stress, including nanoparticle induced toxicity [28]. Similarly, GroEL [29], a chaperone that works closely with DnaK in maintaining proteostasis, was reportedly upregulated by *Campylobacter jejuni* cells in response to ZnO nanoparticle toxicity [30].

Gold nanoparticles have been shown to reverse heat-induced aggregation of proteins in vitro [19]. Therefore DnaK and gold nanoparticles both exhibit protein stabilising function. However, some studies suggest that gold nanoparticles may induce toxicity in bacteria [11]. The effects of gold nanoparticles on *E*. *coli* cell growth, and in particular their effects on the integrity of the *E*. *coli* proteomic constituents are not fully established. In addition, the role of DnaK in *E*. *coli* cells subjected to gold nanoparticle toxicity remains to be explored. In the current study, we investigated the effects of gold nanoparticles on *E*. *coli* BB1553 cells that possess a deleted *dnaK* gene [23]. The AuNPs were internalised by the *E*. *coli* cells. The particles further aggregated inside the cells complexing with cytosolic material. The DnaK deficient *E*. *coli* BB1553 (DnaK^-^) cells that internalised the AuNPs exhibited features such as plasma membrane pulled off the cell wall and filamentation. On the other hand, cells in which DnaK was re-introduced (DnaK^+^) were less susceptible to the toxicity that was induced by the AuNPs. Of particular note, was that *E*. *coli* cells expressing DnaK exhibited distinct zones to which the nanoparticles were restricted in the cell. We also investigated the effects of the AuNPs on the solubility of *E*. *coli* proteins as well as their effects on the function of a DnaK/Hsp70 homologue, *Plasmodium falciparum* Hsp70 [25][31]. To the best of our knowledge this is the first study that demonstrates a role for DnaK in conferring cytoprotection to *E*. *coli* cells exposed to gold nanoparticles.

## Results

### Synthesis and Characterisation of Citrate Capped Gold Nanoparticles

**Fig 1:**
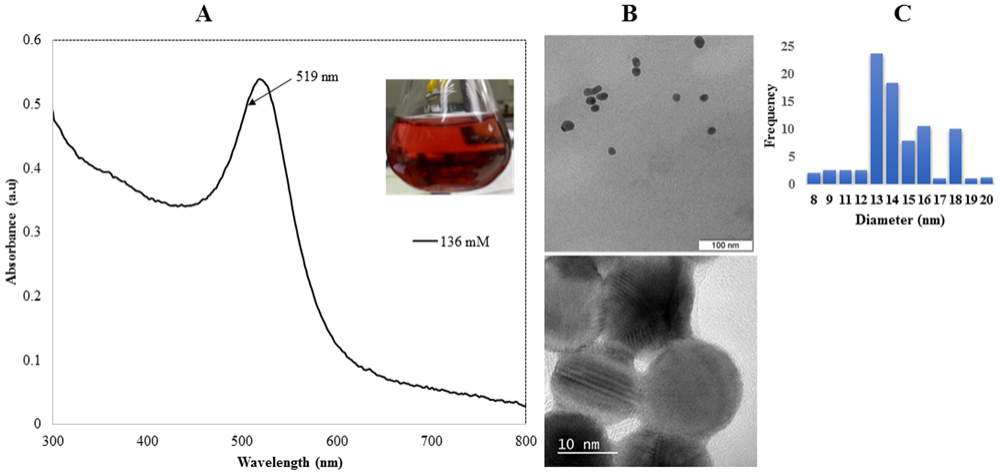
TEM and HRTEM images of synthesised citrate-coated gold nanoparticle 0.3 mM gold salt was reduced with 136 mM tri-sodium citrate. (**A**) Absorption spectra of citrate capped AuNPs; the insert shows the red wine suspensions obtained. (**B**) TEM images of the citrate AuNPs (top panel), and HRTEM images of AuNPs (lower panel). The presence of visibly defined lattice fringes confirmed the crystal morphology of the nanoparticles produced. (**C**) Bar graph representing the frequency of the citrate capped AuNPs by size.

The absorption spectrum of the citrate-coated AuNPs synthesised is shown on Fig. 1A. The absorption peak at 519 nm (Fig. 1A), is assigned to the surface plasmon absorption spectrum of AuNPs which ranges between 510–530 nm [32]. The spectrum appeared narrow suggesting a narrow range of the diameters of the particles obtained. High citrate concentrations are known to limit the growth of nanoparticles [33]. It is possible that the citrate concentration used in this study (136 mM) could have resulted in smaller nanoparticles with a narrow size range. As observed under TEM, the synthesised citrate AuNPs were consistently spherical and mono-dispersed (Fig. 1B, top panel). The representation of the nanoparticles obtained based on their sizes is shown (Fig. 1C). The nanoparticles exhibited an average diameter of 16.2 nm with a standard deviation of ± 2.59 nm. The HRTEM image showed distinctly defined lattice fringes confirming the crystalline nature of the particles (Fig. 1B, lower panel). At higher resolution, the presence of a layer around the particles was also evidently visible suggesting the presence of a citrate coating on the nanoparticle surface.

### *E*. *coli* cells with a Deleted *dnaK* Gene are Sensitive to Toxicity Induced by Citrate-Coated Gold Nanoparticles

**Fig 2:**
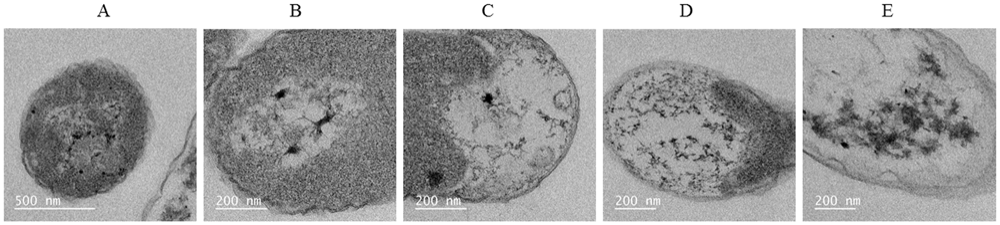
Citrate-AuNPs induce cytotoxicity in *E*. *coli* DnaK deficient cells. TEM images showing *E*. *coli* DnaK^-^ cells exposed to 40 μgmL^-1^ AuNPs. The images show various degrees of cell damage: (A) cell that has internalised AuNPs; (B) AuNPs complexed with cytosolic material; (C) cell matrix disintegrating in the presence of AuNPs; (D) extensive cytosolic disintegration; (E) dead cell showing membrane pooled off the cell.

**Fig 3:**
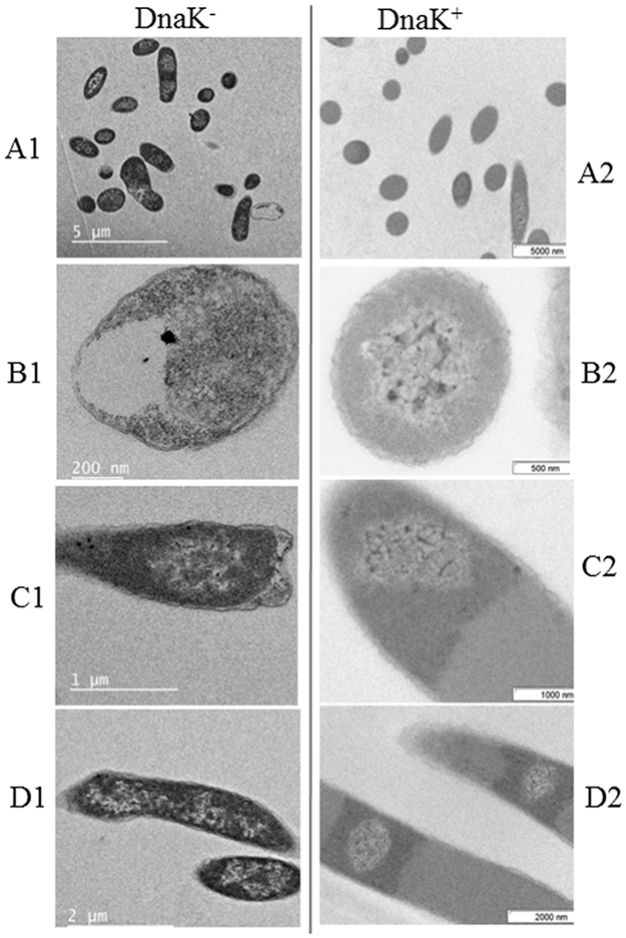
Comparative analysis of the effects of citrate AuNPs on *E*. *coli* DnaK^-^ and *E*. *coli* DnaK^+^ cells. TEM images showing *E*. *coli* DnaK^-^ and *E*. *coli* DnaK^+^ cells exposed to citrate-AuNPs. A comparative population overview of *E*. *coli* DnaK^-^ cells cultured in the presence of AuNPs is shown (panel A1) versus *E*. *coli* DnaK^+^ cells cultured under similar conditions (panel A2). Panels B1-B2 and C1-C2 illustrate comparative morphological features of *E*. *coli* DnaK^-^ cells versus *E*. *coli* DnaK^+^ cells, respectively. AuNP aggregates are seen as black spots inside the cells. Note the evident delineation zones in panels C2 and D2, associated with *E*. *coli* DnaK^+^ cells that are missing in *E*. *coli* DnaK^-^ cells (panels C1and D1).

*E*. *coli* DnaK^-^ cells that were cultured in the presence of 40 μgmL^-1^ citrate AuNPs exhibiting various stages of cell deformity were observed by TEM (Fig. 2). It has been previously demonstrated that AuNPs are readily internalised by *E*. *coli* cells [10][11]. The internalisation of AuNPs by the *E*. *coli* DnaK^-^ cells was evident (Fig. 2) and the nanoparticles appeared to complex with cytoplasmic material, leading to cell death. Comparative TEM views of *E*. *coli* DnaK^-^ cells versus *E*. *coli* DnaK^+^ cells cultured in the presence of AuNPs show distinct features between the two groups of cells (Fig. 3). While a few nanoparticles were internalised as single entities, most of the AuNPs appeared in agglomerations in the cells. Based on TEM images, there is evidence that the AuNPs complexed with the cytosolic material both in DnaK^-^ and DnaK^+^ *E*. *coli* cells. Both *E*. *coli* DnaK^+^ cells and *E*. *coli* DnaK^-^ cells exposed to AuNPs at 30°C had cells of normal shape and size as well as some that had filamented (Fig. 3, panels A1; A2). However, a higher proportion of filamented cells were observed in the *E*. *coli* DnaK^-^ sample. Filamentation of bacteria is associated with cellular stress and DnaK deficiency promotes this phenomenon [23]. It is possible that the filamentation observed in some cells from the *E*. *coli* DnaK^+^ cell population may have been induced by AuNP toxicity. We also noticed that a large proportion of *E*. *coli* DnaK^-^ cells had invaginations and their plasma membranes pulled off from the cell wall (Fig. 3; panels B1-B2; C1-C2). However, a large proportion of *E*. *coli* DnaK^+^ cells exhibited normal cellular integrity. We also noticed distinct “zones” that confined the nanoparticles to specific sites within the cytosol of *E*. *coli* DnaK^+^ cells (Fig. 3; panels C1-C2; D1-D2). It is not clear what role DnaK played in the development of these distinct structural features. However, it is evident that DnaK was involved in the development of these features, as *E*. *coli* DnaK^-^ cells did not exhibit similar features. As a result, most of the *E*. *coli* DnaK^-^ cells did not appear to restrict the localisation of the AuNP nucleation zones to specific locations within the cell (Fig. 3; panels D1-D2).

### Effect of Gold Nanoparticles on the Expression and Solubility of Proteins in *E*. *coli* BB1553 Cells Exposed to Gold Nanoparticles

**Fig 4:**
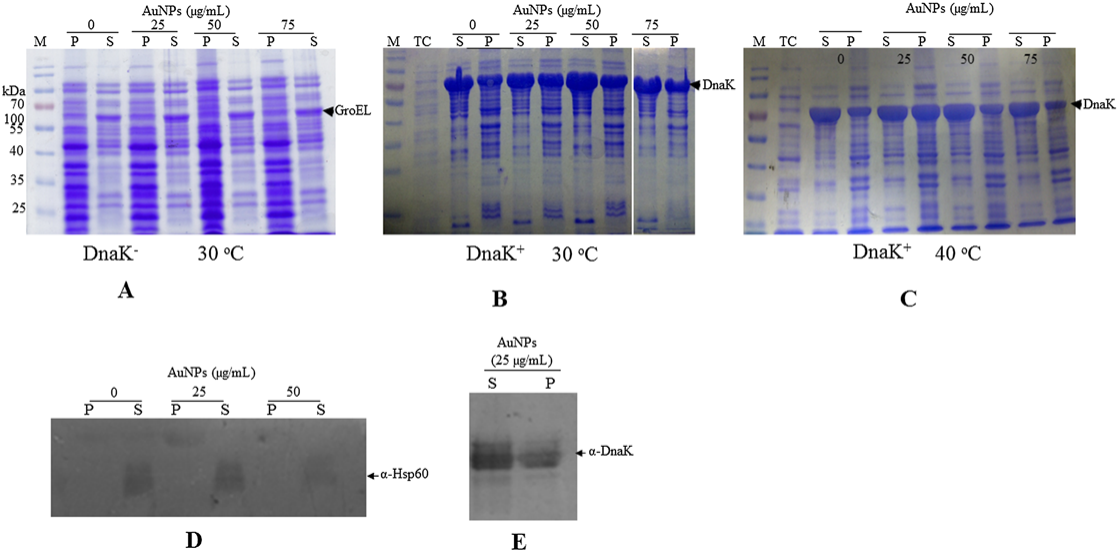
Expression and solubility profiles of *E*. *coli* BB1553 cells exposed to AuNPs. SDS-PAGE analysis representing protein expression and solubility profiles of *E*. *coli* BB1553 cultured in the absence and presence of AuNPs (25–75 μgmL^-1^). Various fractions of *E*. *coli* cells were obtained: (**A**) *E*. *coli* DnaK^-^ cells cultured at 30°C in the absence and presence of variable AuNPs; (**B**) *E*. *coli* DnaK^+^ cells cultured at 30°C in the absence and presence of variable levels of AuNPs; (**C**) *E*. *coli* DnaK^+^ cells cultured at 40°C in the absence and presence of variable levels of AuNPs. Lanes representing total cell lysate (TC); cell pellet (P) and soluble (S) fractions, respectively, are shown. Western blot analyses were conducted to confirm production of heterologously expressed DnaK (**D**); and endogenous GroEL (**E**). Pellet and soluble fractions of *E*. *coli* BB1553 that had been exposed to AuNPs at the given concentrations were sampled for the Western analyses.

*E*. *coli* DnaK^+^ and *E*. *coli* DnaK^-^ cells were exposed to citrate AuNPs at variable levels (25–75 μgmL^-1^) and incubated at 30°C. Based on the SDS-PAGE data, *E*. *coli* DnaK^-^ cells exposed to AuNPs had a more enriched pellet fraction compared to that isolated from cells that had not been exposed to AuNPs (Fig. 4A). This suggests that the presence of AuNPs marginally promoted the aggregation of proteins in *E*. *coli* DnaK^-^ cells. GroEL, a chaperone that cooperates with DnaK in *E*. *coli* was expressed in *E*. *coli* DnaK^-^ cells cultured at 30°C in the absence and presence of AuNPs. However, its production was more enhanced in *E*. *coli* DnaK^-^ cells in the presence of AuNPs. The over-production of GroEL in *E*. *coli* DnaK^-^ cells exposed to increasing levels of AuNPs further testifies that the presence of AuNPs further subjected the cells to additional stress. DnaK was evidently over-produced in *E*. *coli* DnaK^+^ cells (Fig. 4B). As expected, GroEL was not over-expressed in *E*. *coli* DnaK^+^ cells. This is in agreement with DnaK playing the most prominent role in protein folding and thus GroEL was over-expressed in the absence of DnaK as a compensatory measure [34]. The solubility profile of the proteomic constituents of *E*. *coli* DnaK^+^ cells cultured in the presence and absence of AuNPs that were incubated at 30°C was similar (Fig. 4B). This suggests that the presence of DnaK reduced the detrimental effects of the AuNPs. A batch of *E*. *coli* DnaK^+^ cells cultured in the presence of AuNPs was subjected to heat stress at 40°C. We had expected that the possible detrimental effects of the AuNPs on the proteome of *E*. *coli* DnaK^+^ cells would be more apparent at higher temperatures. However, based on the SDS-PAGE data, there was no difference in the protein solubility profile in *E*. *coli* DnaK^+^ cells cultured at 40°C in the presence and absence of AuNPs (Fig. 4C). Altogether, the findings suggest that AuNPs at comparable levels (25–75 μgmL^-1^) promoted protein aggregation in *E*. *coli* cells that lacked DnaK and that restoration of DnaK in these cells conferred cytoprotection. The expression of endogenous GroEL by *E*. *coli* DnaK^-^ cells that were exposed to various levels of AuNPs was validated by Western blotting (Fig. 4D). Similarly, the expression of DnaK by *E*. *coli* DnaK^+^ cells exposed to AuNPs was confirmed by Western blot analysis (Fig. 4E). The Western blot data suggests that GroEL occurred in the soluble fraction of the cells, whilst DnaK was spilt between the soluble and insoluble fraction of the cells (Fig. 4D-E).

### Effect of Citrate-Coated Gold Nanoparticles on Heat-Induced Protein Aggregation of Malate Dehydrogenase Depends on their Concentration

**Fig 5:**
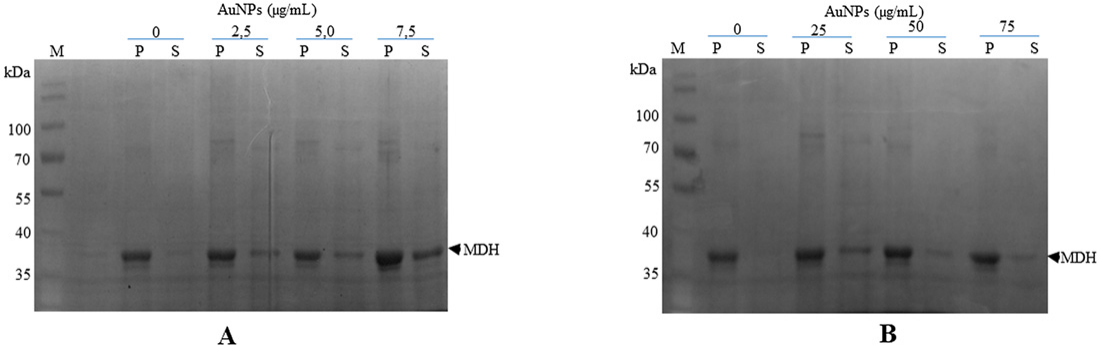
Citrate-coated gold nanoparticles suppress malate dehydrogenase aggregation in a concentration dependent manner. (**A**) 1 μM MDH was suspended in assay buffer in the absence or presence of various levels of AuNPs (2.5–7.5 μgmL^-1^). (**B**) The assay was repeated in the presence of 10 fold higher levels of AuNPs (25–75 μgmL^-1^). The suspensions were subjected to heat stress at 48°C for 20 minutes. The soluble fraction (S) was separated from pellet fraction (P) by centrifugation. Samples were analysed by SDS-PAGE analysis.

In order to further investigate the effect of citrate-coated AuNPs on the integrity of protein under heat stress conditions we exposed MDH, an aggregation prone protein, to heat stress at 48°C in the absence and presence of the citrate-coated AuNPs at low concentration range (2.5–7.5 μgmL^-1^) and ten-fold higher levels (25–75 μgmL^-1^). In the absence of AuNPs nearly all the MDH subjected to heat stress aggregated (Fig. 5A-B; lane “0”). However, the aggregation of MDH was suppressed in the presence of AuNPs at lower concentration range (2.5–7.5 μgmL^-1^; Fig. 5A). On the other hand AuNPs at higher levels (25–75 μgmL^-1^) were less effective in suppressing MDH aggregation (Fig. 5B).

**Fig 6:**
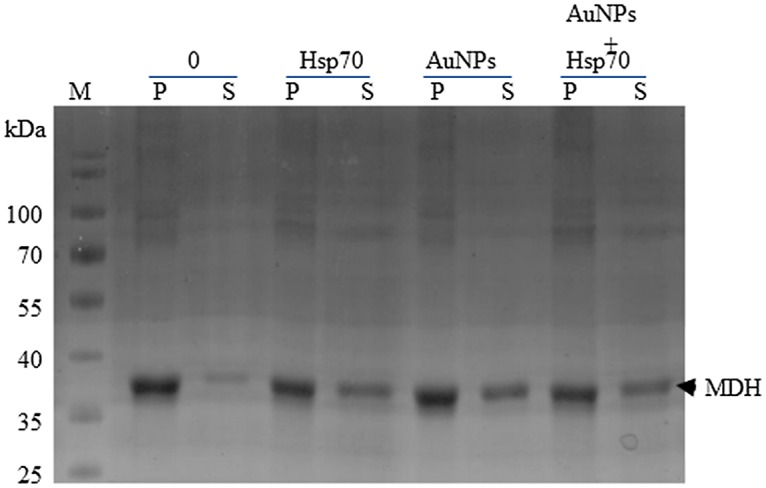
Suppression of MDH aggregation by citrate-coated gold nanoparticles combined with Hsp70. 1 μM MDH was suspended in assay buffer in the absence or mixed with 1.3 μM Hsp70 or AuNPs (10 μgmL^-1^); and a combination of Hsp70 and AuNPs, respectively. The suspensions were subjected to heat stress at 48°C for 20 minutes. The soluble fraction (S) was separated from pellet fraction (P) by centrifugation. Samples were analysed by SDS-PAGE analysis.

Hsp70 proteins act as molecular chaperones that suppress protein aggregation as well as refold misfolded proteins [35]. Previously, we observed that cysteine-coated AuNPs did not interfere with the chaperone function of human Hsp70 [19]. In the current study, we sought to investigate the effect of citrate-coated AuNPs on the function of another DnaK/Hsp70 homologue, *Plasmodium falciparum* Hsp70 [25][31] that is known to possess chaperone function [36]. As expected, the Hsp70 chaperone promoted the retention of MDH in soluble form (Fig. 6; lane “Hsp70”). In addition, the heat-induced aggregation of MDH was significantly suppressed by the citrate AuNPs (Fig. 6; lane “AuNPs”). A mixture of the Hsp70 chaperone and AuNPs also suppressed the aggregation of MDH (Fig. 6; lanes “AuNPs+Hsp70”). This suggests that the AuNPs at this concentration (10 μgmL^-1^) did not interfere with Hsp70 function. These findings suggest that the citrate-coated AuNPs are capable of suppressing MDH aggregation in vitro as we previously observed with cysteine-coated AuNPs [19].

**Fig 7:**
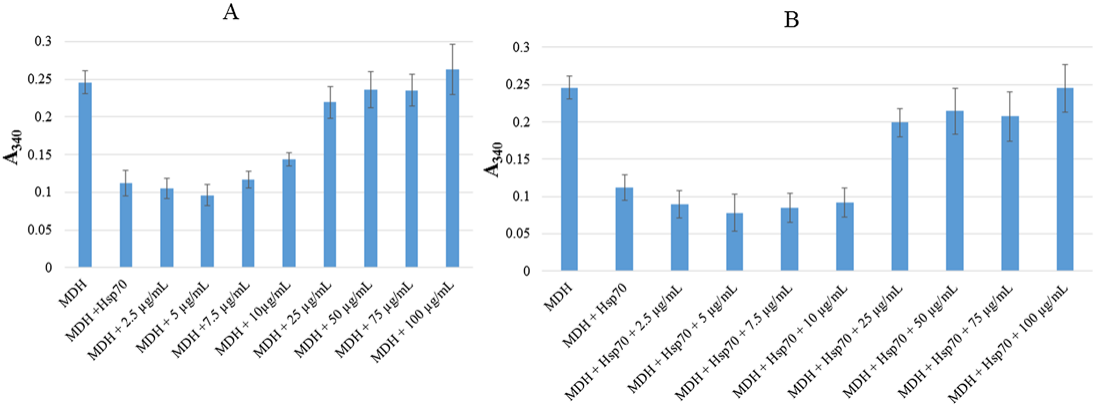
Spectrophotometric analysis for the heat-induced aggregation of MDH in the presence of AuNPs and Hsp70. 1 μM MDH was suspended in assay buffer in the absence or mixed with high concentrations of AuNPs (0–100 μgmL^-1^) (**a**); the assay was repeated in the presence of 1.3 μM Hsp70 (**b**). The suspensions were subjected to heat stress at 48°C for 20 minutes. Absorbance values were measured at 340 nm in triplicates using a 96-well micro titre plate. Data are presented as mean and standard deviations.

To further validate the findings, we monitored aggregation of MDH by taking spectrophotometric readings at 340 nm. The heat-induced aggregation of MDH results in turbidity which could be monitored by taking absorbance readings using a spectrophometer set at A_340_. As expected the Hsp70 chaperone suppressed the aggregation of MDH (Fig. 7). Furthermore, low levels of AuNPs (2.5–10 μgmL^-1^) suppressed the heat-induced aggregation of MDH in a concentration dependent fashion. In agreement with the findings obtained using SDS-PAGE analysis, higher levels (25–100 μgmL^-1^) of the AuNPs were not effective in suppressing the aggregation of MDH (Figs. 5B and 7A). The assay was repeated in the presence of 1.3 μM Hsp70. Hsp70 and the AuNPs at lower levels (2.5–10 μgmL^-1^) complemented each other in suppressing MDH aggregation (Fig. 7B). However, the aggregation of MDH in the presence of higher levels of AuNPs (25–100 μgmL^-1^) could not be suppressed by adding 1.3 μM Hsp70 to the reaction mix (Fig. 7B). This suggests that the citrate-coated AuNPs may have agglomerated at higher concentrations, presenting a surface curvature that compromised folding of MDH.

## Discussion

In this study we investigated the effect of citrate-coated AuNPs on *E*. *coli* cells that lack the gene for the molecular chaperone DnaK. Since AuNPs exhibit chaperone like function in vitro, we sought to investigate their effect on *E*. *coli* cells that lack DnaK as the protein folding pathway of the cells is compromised, making them highly stress susceptible. It is known that *E*. *coli* cells tend to up-regulate DnaK expression in response to stress, including nanoparticle induced toxicity [28]. However, it is not fully understood what role DnaK plays under such conditions. AuNPs have been described as fairly inert and less toxic [37]. However, evidence from the current study and independent studies [8][13][11], suggest that AuNPs exhibit detrimental effects to *E*. *coli* cells. DnaK is a conserved molecular chaperone whose main function is to facilitate protein folding in *E*. *coli* [23]. *E*. *coli* cells that lack the *dnaK* gene exhibit compromised protein folding functions and grow at 30°C and do not thrive at higher temperatures [38]. Because of their stress-susceptibility and their compromised protein folding system, *E*. *coli* BB1553 cells are ideal for studying protein folding in *E*. *coli* [15]. Similar *E*. *coli* cells have also been used to study the effects of antimicrobial agents that target DnaK function [26]. In the current study, *E*. *coli* DnaK^-^ cells cultured at their ambient temperature of growth (30°C) in the presence of AuNPs exhibited growth defects such as plasma membrane that pulled off from the cell wall, leading to cell death. In addition, a high proportion of the cells that survived were filamented, suggesting that they were under severe stress. The defects were alleviated in The *E*. *coli* DnaK^+^ cells. In addition, *E*. *coli* DnaK^+^ cells that internalised the AuNPs exhibited distinct structures that appeared to form around the AuNP agglomerates which restricted these nucleation sites to distinct zones in the cytosol (Fig. 3, panels C2; D2). These structures were evident only in The *E*. *coli* DnaK^+^ cells. This study provides evidence for a role of DnaK in alleviating toxicity induced by the AuNPs in *E*. *coli*.

Growing evidence suggesting that AuNPs are capable of suppressing stress-induced protein aggregation [19][20] has raised prospects for the application of gold nanoparticles as agents for the treatment of conditions related to protein misfolding such as Alzheimer’s disease [22]. However, there is lack of evidence regarding the effects of gold nanoparticles on protein integrity in vivo. Since cysteine-coated AuNPs were previously shown to have protein aggregation suppressing functions [19], in the current study, we investigated if this feature is shared by citrate-coated AuNPs. In addition, because of their protein aggregation suppression function, AuNPs share this functional feature (chaperone-like function) with molecular chaperones amongst them, DnaK/Hsp70 [25][27][36].

To understand the effect of AuNPs on the integrity of the *E*. *coli* BB1553 proteome, we cultured the cells in the absence and presence of increasing levels of AuNPs. The cells were incubated at their ambient growth temperature (30°C) and another batch was exposed to a non-permissive temperature of 40°C. We observed that The *E*. *coli* DnaK^-^ cells incubated at 30°C in the absence of AuNPs exhibited a proteomic solubility profile that resembled that of cells that were exposed to AuNPs (Fig. 4). The *E*. *coli* DnaK^-^ cells also expressed GroEL, a molecular chaperone that is known to facilitate protein folding [34]. Since GroEL was not over-expressed by *E*. *coli* DnaK^+^ cells, GroEL over-production must have served as compensation for lack of DnaK function. On the other hand, The *E*. *coli* DnaK^+^ cells exhibited similar protein solubility profiles irrespective of whether they were cultured in the absence or presence of the AuNPs. Thus DnaK prevented the proteomic constituents of *E*. *coli* BB1553 cells exposed to AuNPs from aggregating even when the cells were exposed to an elevated growth temperature of 40°C.

**Fig 8:**
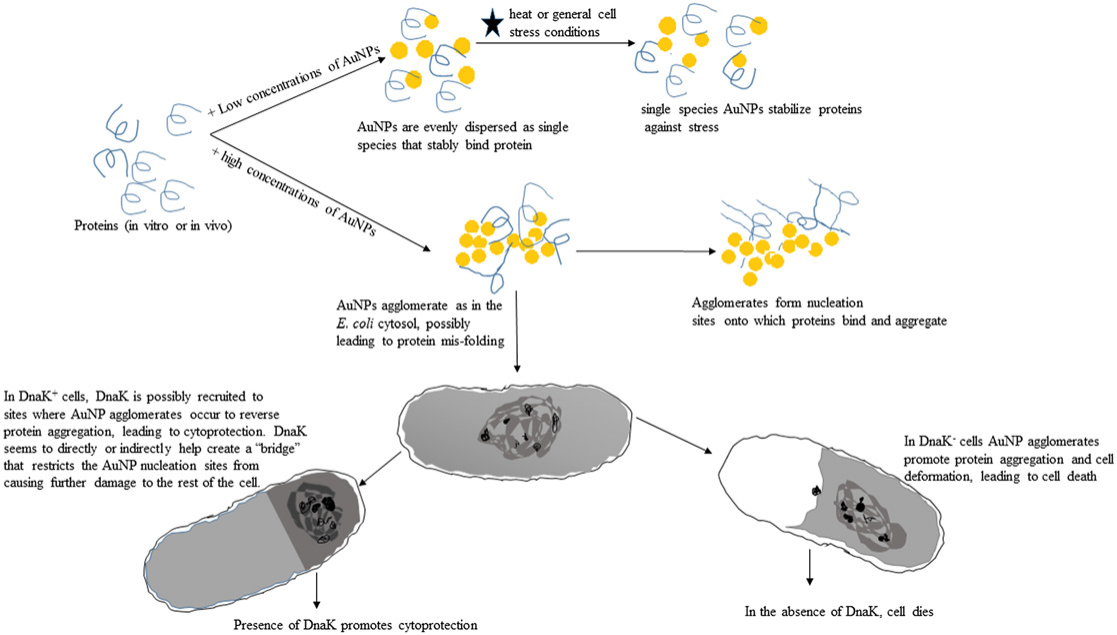
Proposed model illustrating the effects of citrate-coated gold nanoparticles in vitro and in *E*. *coli* cells. The model describes the proposed effects of single species versus agglomerated species of citrate-gold nanoparticles on the integrity of proteins in vitro and in *E*. *coli* cells that are deficient of DnaK and in which DnaK function is restored.

To further confirm the effect of citrate coated AuNPs on protein integrity, we exposed an aggregation prone protein, MDH, to heat stress in the absence and in the presence of variable levels of AuNPs (Figs. 5 and 6). We observed that at lower order levels (2.5–10 μgmL^-1^), AuNPs effectively suppressed heat-induced aggregation of MDH. However, at higher order levels (25–100 μgmL^-1^), AuNPs were not effective in suppressing the heat-induced aggregation of MDH (Figs. 5B and 7A-B). AuNPs at higher concentrations tend to agglomerate [11][38]. Under such conditions, they possibly present a surface curvature that is detrimental to protein stability. It is interesting to note that the addition of Hsp70 to MDH in the presence of high levels of AuNPs did not result in reduction of MDH aggregation (Fig. 7B). This suggests that at high levels, AuNPs may have promoted protein aggregation that Hsp70 failed to reverse in vitro. However, in the cell, Hsp70 is more effective in reversing aggregates as it cooperates with other molecular chaperones [35]. This could explain why the restoration of DnaK in *E*. *coli* BB1553 cells promoted cell recovery even though the internalised AuNPs appeared agglomerated. We hypothesize that the AuNP nucleation sites created a platform that promoted protein misfolding and aggregation (Fig. 8). Since DnaK has a propensity to bind to misfolded protein [14], it is possible it was recruited to the nucleation sites by misfolded proteins, leading to the formation of the distinctly dense structures viewed under TEM (Fig. 3, panels C2, D2; Fig. 8). Subsequently, DnaK, possibly in cooperation with other chaperones in the cell effectively reversed protein aggregation, leading to cell survival. In the absence of DnaK, we propose that the agglomerations formed by the AuNPs in the absence of DnaK promoted irreversible protein aggregation. This is supported by the observation that *E*. *coli* DnaK^-^ cells exposed to AuNPs had a higher proportion of proteins in the pellet fraction, compared to cells that were not exposed to AuNPs. On the other hand, *E*. *coli* DnaK^+^ cells that were exposed to AuNPs exhibited similar protein solubility pattern to that observed in *E*. *coli* DnaK^+^ cells that were not exposed to AuNPs (Fig. 4).

## Materials and Methods

### Synthesis of Citrate-Coated Gold Nanoparticles

We synthesized water soluble gold nanoparticles using a previously described citrate reduction method [32][39] with slight modifications. 0.3 mM of HAuCl_4_.3H_2_O was heated to 95°C followed by the drop-wise addition of 136 mM sodium citrate tribasic dihydrate (C_6_H_5_Na_3_O_7_.2H_2_O) under constant stirring. Colour changes in the reaction mixture were noted upon the addition of sodium citrate until a wine red colour was observed. The reaction proceeded for approximately 10 minutes and was allowed to cool to room temperature with continued stirring. Samples were collected and prepared for characterization using ultraviolet visible spectrophotometry (UV-VIS), transmission electron microscopy (TEM) and high resolution transmission electron microscopy (HRTEM).

### UV-Spectrophotometry, Transmission Electron Microscopy and High Resolution Transmission Electron Microscopy

A Spectroquant Pharo 300 was used to measure the UV spectra of these citrate-AuNPs in the range 200–1000 nm using a 1 cm path length cuvette. To image the nanoparticles, a JEOL 1010 TEM with an accelerating voltage of 100 kV, Megaview III camera and Soft Imaging Systems iTEM software was used. The images provided information on the morphology, size and dispersion of the AuNPs. A JEOL HRTEM with accelerating voltage of 200 kV, Megaview III camera and Soft Imaging Systems iTEM software was used to image the nanoparticles at high magnifications to assess the crystal nature of the nanoparticles.

### Assessment of the Effects of Gold Nanoparticles on *E*. *coli* BB1553 Cells

*E*. *coli* BB1553 (MC4100 Δ*dnaK*52::*Cm* ^*R*^ *sidB1)* cells lack the *dnaK* gene [23] and, therefore, possess compromised protein folding capabilities. We previously used the same cells in another study [25]. The cells were kindly provided by Dr. Bernd Bukau (University of Heidelberg).

A construct expressing DnaK (pQE60/DnaK; originally provided by Dr W. Burkholder, Stanford University) and a neat vector plasmid (pQE60) were transformed into *E*. *coli* BB1553 cells. For convenience, we refer to *E*. *coli* BB1553 that were transformed with vector plasmid as ‘*E*. *coli* DnaK^-^’ and the cells that were transformed with the DnaK construct as ‘*E*. *coli* DnaK^+^’. Following transformation, a single colony was inoculated into 2YT broth (16 g of tryptone powder, 10 g of yeast extract powder and 5 g of sodium chloride in 1000 mL of double distilled water) supplemented with 35 μgmL^-1^ chloramphenicol and 100 μgmL^-1^ ampicillin and left to grow overnight. Next, 5 μL of inoculum from the overnight cultures was transferred into 45 mL of fresh 2YT broth, supplemented with 35 μgmL^-1^ chloramphenicol and 100 μgmL^-1^ ampicillin. The cells were incubated at 30°C with shaking to optical density (OD_600_) of 0.6. Citrate gold nanoparticles were added to the culture at a final concentration of 40 μgmL^-1^ and incubated for 12 hours. The cells were pelleted by centrifugation at 14000 rpm and the pellets were washed 5 times with phosphate buffered saline (PBS) (pH 7.5) at 5000 rpm.

The cells were then prepared for TEM analysis by initial primary fixation in 500 μL of buffered 2.5% glutaraldehyde for 24 hours. Washing of the cell pellets three times for 5 min with phosphate buffer was followed by post fixing for 1 hour in 0.5% osmium tetroxide. Three additional wash steps were conducted each lasting 5 min using PBS (pH 7.5). Dehydration of the cells was conducted by re-suspending them two times in 30%, two times in 50% and two times in 75% acetone for 5 minutes each time. The cells were further re-suspended twice in 100% acetone for 10 minutes each time. A 50:50 ratio of resin (4.1g ERL 4221, 5.9 g NSA, 1.43 DER 736 and 0.1 g DMAE) and acetone was used to infiltrate the samples for 4 hours after which they were left in whole resin for 24 hours. The specimens were subsequently polymerised in whole resin for 8 hours at 70°C in an oven. Thin sections were cut and specimens were mounted on copper grids, stained and viewed on a JOEL 1010 TEM.

### Analysis of the expression and solubility patterns of proteins in *E*. *coli* BB1553 cells exposed to citrate coated gold nanoparticles

Samples from *E*. *coli* BB1553 cells that were cultured under conditions as described previously in this study, were taken after 24 hours of growth to investigate the expression and solubility status of proteins by *E*. *coli* BB1553 cells exposed to AuNPs. The *E*. *coli* cells were pelleted by centrifuging at 3500 rpm for 20 minutes at 4°C and resuspended in 5 ml lysis buffer (0,01 mM Tris,pH 7.5; 10 mM Imidazole,containing 1 mM phenylmethylsulfonyl fluoride (PMSF) and 1 mgmL^-1^ of lysozyme). The cells were thawed rapidly and mildly sonicated following an overnight storage at -80°C. Soluble and insoluble fractions were seperated by centrifuging at 5000 rpm at 4°C for 20 minutes. These fractions were resolved by SDS-PAGE at 12% gel density. Western blotting analysis was done to confirm expression of DnaK and GroEL proteins, using anti-DnaK (Stressgen) and anti-Hsp60 antibodies respectively. The anti-Hsp60 antibodies were kindly donated by Prof. Alister Craig, Liverpool University, UK.

### Effect of gold nanoparticles on the solubility of heat-treated malate dehydrogenase

Aggregation of MDH was investigated in the presence (2.5–100 μgmL^-1^) and absence of AuNPs, following a previously described protocol [19]. Briefly, 1 μM of porcine heart MDH (Sigma-Aldrich, St. Louis, MO) was suspended in assay buffer (20 mM tris, pH 7.4; 100 mM NaCl [19][36]. AuNPs were added to each reaction mix to final concentrations ranging from 2.5 to 100 μgmL^-1^. As a positive control, 1.3 μM of *Plasmodium falciparum* Hsp70, a homologue of DnaK [36] was included in place of AuNPs. An additional assay was set-up to investigate the effect of AuNPs on the chaperone activity of Hsp70. In the latter set-up, AuNPs and Hsp70 were both added to the reaction mix. The suspensions were incubated at 48°C for 20 minutes. To separate soluble from insoluble fractions, the reaction mix was centrifuged at 14 000 rpm for 10 min at 4°C. MDH in the soluble and pellet fraction was resolved by sodium dodecyl sulphate gel electrophoresis (SDS-PAGE). As a confirmation assay, the heat-induced aggregation of MDH was further assessed by taking absorbance readings every 5 minutes for 60 min using a Biotech ELX 800 plate reader set at a temperature of 48°C.

## Conclusions

Altogether our findings demonstrated that citrate-coated AuNPs are internalised in *E*. *coli* cells mainly as agglomerates. We hypothesize that single citrate-coated gold nanoparticle entities promote protein stability, while highly concentrated particles agglomerate to promote protein aggregation. Since the citrate-coated AuNPs were internalised mainly in agglomerated form, they promoted protein aggregation, leading to death of *E*. *coli* DnaK^-^ cells. We suppose that DnaK alleviated cytotoxicity through its ability to reverse protein aggregation. However, further evidence for the direct role of DnaK in alleviating cytotoxicity of *E*. *coli* cells is required. Furthermore, since GroEL was upregulated in *E*. *coli* DnaK^-^ cells, it remains to be understood how these two prominent molecular chaperones of *E*. *coli* assist cells survive stress.
